# Supplementary material for: Rapid and Visual Screening of Virus Infection in Sugar Beets Through Polerovirus-Induced Gene Silencing
Source: Viruses. 2024 Nov 23;16(12):1823. doi: 10.3390/v16121823 (PMC11680160; doi:10.3390/v16121823)
Supplement: Supplementary file 1 [file viruses-16-01823-s001.zip › viruses-3282479-Supplementary Files.pdf]

Table S1. List of oligonucleotides used for the construction of the BMYV-SUL infectious clone, for RT-PCR reactions and northern blot probes.

Primer names	Primer sequences
SUL-pBS_F	TTGATGAGGTTAATCTTTTGGATGACCACTTGGTGGATGTTCTTTTGGATTCT GCACCTTTCCCAATCATCACAGTCAAGCCCG
35S_R	GGTCTTCTGAGACTGTATCTTTG
M13_F	GTAAAACGACGGCCAGT
SUL-BMYV_R	ACATCCACCAAGTGGTCATCCAAAAGATTAACCTCATCAACATACAGAATTCC TCTCATCAGAACCTTGGGTCAGGGTTTGTGCC
BMYV_CT_rev2019	ACACCGAAGTGCCGTAGGGAG
BMYV_5300_F	CCCCGGATATTCCAAGGAAGAC
BMYV_CT_rev2021	TTATCCCAGCGTAAAACACTCG
SUL-F	GAGGAATTCTGTATGTTGAT
SUL-R	GCAGAATCCAAAAGAACATC
miR160	TGGCATACAGGGAGCCAGGCA
BMYV_NB_F	AAGACAATCTCGCGGGAAGT
BMYV_NB_R	TGGGAATACCATCGAGAATC

S1

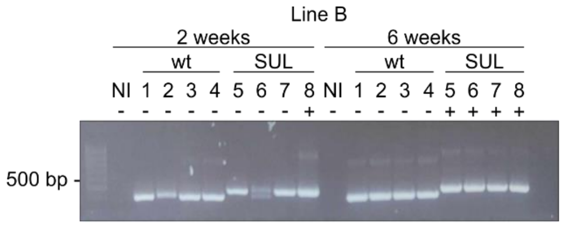

Figure S1. Analysis of viral RNAs produced by BMYV infection over time. RT-PCR analysis of non-agroinoculated (NI), wild-type (wt) or recombinant (SUL) BMYV agroinoculated plants of line B, using oligonucleotides flanking the inserted SUL fragment site. Bands of 402 bp and 472 bp were respectively expected for wild-type BMYV and recombinant BMYV-SUL constructs. Asymptomatic and symptomatic plants are respectively annotated by a minus (-) or a plus (+) sign.

S2

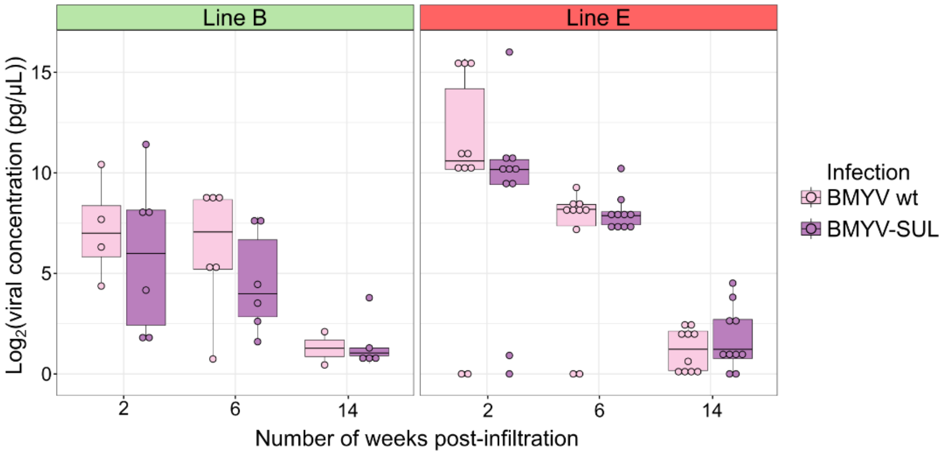

Figure S2. **Persistence of BMYV-SUL induced VIGS in sugar beets.** Estimated viral load of infected plants, over the 14 weeks is here represented as box plots, pink for wild-type BMYV and purple for recombinant BMYV-SUL.
